# Myocarditis as the Initial Presentation of a Refractory Adult-Onset Still’s Disease

**DOI:** 10.7759/cureus.29821

**Published:** 2022-10-01

**Authors:** Nuno Pina Gonçalves, Maria Helena Lourenço, Francisco Albuquerque, Maria J Gonçalves, Sandra Falcão

**Affiliations:** 1 Rheumatology, Hospital de Egas Moniz, Centro Hospitalar Lisboa Ocidental, Lisbon, PRT; 2 Cardiology, Hospital de Santa Cruz, Centro Hospitalar Lisboa Ocidental, Lisbon, PRT; 3 Rheumatology, Hospital de Loures, Lisbon, PRT

**Keywords:** severe, refractory disease, biologic therapies, peri-myocarditis, adult onset still's disease (aosd)

## Abstract

We present a case of adult-onset Still’s disease, a rare disease that presented with a rare complication, myocarditis. After initial therapy with prednisolone 1 mg/kg/day, the patient experienced dyspnea due to severe pleural effusion and palpitations on account of new-onset supraventricular tachycardia. Therapy with three consecutive pulses of 1000 mg of methylprednisolone and anakinra 100 mg/day proved to be effective, with a progressive resolution of all symptoms. However, at three months follow-up, oligoarthritis recurred along with high serum ferritin. Secondary failure to anakinra was assumed, and a switch to tocilizumab 162 mg was made. Nevertheless, arthralgia of the wrists and knees as well as high serum ferritin still persisted after two months of therapy. Chronic disease was assumed, and the patient switched to canakinumab 4 mg/kg/dose q4week, and a complete resolution of symptoms occurred with normalization of inflammation markers. Follow-up cardiac magnetic resonance showed a complete resolution of heart involvement.

## Introduction

Adult-onset Still’s disease (AOSD) is a very rare multisystemic inflammatory disease mainly characterized by fever, arthritis, and evanescent rash [1]. Although rare, myocarditis is a potentially fatal complication that can lead to arrhythmias, heart failure, and cardiac tamponade. We present a case of AOSD complicated by myopericarditis and supraventricular tachycardia, which was initially responsive to therapy, but with a chronic course later on.

## Case presentation

A male patient in his early 40s, without any priors, started a clinical picture of high fever (42.5ºC), headache, sore throat, abdominal discomfort, myalgia, polyarthralgia (shoulders, wrists, and knees), and pink nonpruritic rash, initially on the trunk with progression to the arms and legs, which was worsened by fever spikes. He was initially admitted to the emergency room and suspected of having Lyme disease. Although blood cultures were negative, two series of antibiotics were made, namely, ceftriaxone and doxycycline as well as ciprofloxacin and metronidazole, posteriorly. Hydrocortisone 200 mg/day was also administered for three consecutive days. The patient was submitted to a colonoscopy with biopsies, with no pathological findings. A presumptive diagnosis of Crohn’s disease was made, and the patient was discharged with corticosteroids and mesalazine.

**Figure 1:**
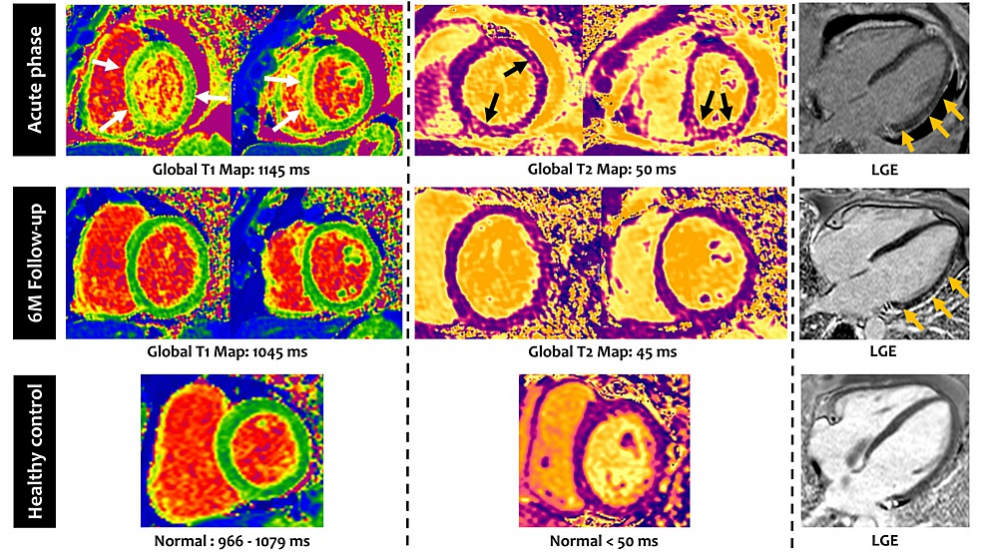
Cardiac magnetic resonance findings in the acute phase and at six months follow-up Cardiac magnetic resonance findings in the acute phase (upper panel) showing global and regional (white and black arrows) increase in T1 and T2 values, reflecting myocardial edema, and mild subepicardial LGE (orange arrows), a marker of myocardial fibrosis. Also, note the presence of pericardial effusion (represented as black in the LGE image). At six months follow-up (middle panel), there was a resolution of the myocardial edema and pericardial effusion while LGE became more prominent (orange arrows). In the lower panel, there is a healthy subject with normal T1 and T2 values and no LGE for comparison. LGE: Late gadolinium enhancement.

After two weeks, he was readmitted due to new-onset chest pain and worsening polyarthralgia. Clinical electrocardiography revealed an ST elevation in I, II, AvL, AvF, and V2-V6 leads. Acute myopericarditis was evidenced by a cardiac magnetic resonance (MRI), showing edema and interstitial fibrosis of the myocardium (Figure 1). Relevant findings from the extensive workup, such as marked neutrophilic leukocytosis, hyperferritinemia, elevated C-reactive protein (CRP) and erythrocyte sedimentation rate (ESR), and high troponin I, are found in Table 1. Coronary heart disease was promptly excluded, and the patient was immediately transferred to the rheumatology ward. At admission, the patient presented bilateral basal crackles and polyarthritis of the shoulders, elbows, wrists, and knees, although with no major functional limitation. Relevant findings of the laboratory workup at the ward admission, such as maintenance of previous findings, with new onset of normochromic normocytic anemia and elevation of liver enzymes, are shown in Table 1. Complete viral serologies and immunological study were negative. The combination of the clinical picture, the elevation of inflammatory markers with very high ferritinemia, and the exclusion of other diseases permitted the diagnosis of AOSD, and the patient started treatment with prednisolone 1 mg/kg/day. However, five days later, there was a recurrence of fever along with new-onset resting dyspnea and incapacitating polyarthritis of the shoulders, wrists, knees, and ankles. A chest x-ray revealed a severe pleural effusion. A thoracentesis was performed, and 100 mL of inflammatory sterile pleural effusion was drained. Meropenem was prescribed in addition to one pulse of 1 g of methylprednisolone for three consecutive days.

**Table 1:** Laboratory findings at the emergency room and rheumatology ward admissions Arrows facing up (↑) indicate a value above the normal range, and the arrows facing down (↓) indicate a value below the normal range. AST: Aspartate transaminase; ALT: Alanine transaminase.

Blood-test	Admission to the emergency room	Admission to the rheumatology ward
Hemoglobin	-	10.5 g/L (↓)
Leukocytes	28.63 x 10^9^/L (88.2% of neutrophils) (↑)	24.07 x 10^9^/L (↑)
Ferritin	31714 µg/L (↑)	
C-reactive protein	27.38 mg/dL (↑)	34.4 mg/dL (↑)
Erythrocyte sedimentation rate	120 mm/h (↑)	120 mm/h (↑)
Troponin I/T	94.3 pg/mL/- (↑)	-/12 ng/mL (↑)
Nt-proBNP	-	528 pg/mL (↑)
Liver enzymes (AST/ALT)	-	44 U/L/33U/L (↑)

On day 6 of admission, the patient experienced palpitations. Clinical examination revealed a heart rate of 250 bpm, and the patient was diagnosed with supraventricular tachycardia. He was admitted to an intensive care unit and was administered adenosine, which effectively reversed the dysrhythmia. A refractory disease was assumed, and anakinra 100 mg/day and methotrexate 15 mg/week were started. In the following days, there was a progressive resolution of dyspnea, pleural effusion, and polyarthritis. At three months follow-up, oligoarthritis recurred (in the wrists and right knee) with high ferritin (496 µg/L). Due to secondary failure to anakinra, synoviorthesis of the affected joints were performed, and tocilizumab 162 mg s.c. e.o.w. was prescribed. After two months of tocilizumab treatment, persistent arthralgia of the wrists and knees, with morning stiffness and maintained elevated ferritin, prompted a switch to canakinumab 4 mg/kg/dose every four weeks. Complete disease remission was achieved with canakinumab 4 mg/kg/dose, and ferritin levels returned to normal range values. A follow-up cardiac MRI was performed six months after discharge, showing resolution of the myopericarditis, without any sequelae (Figure 1). The patient returned to his daily activities without any restrictions and remains asymptomatic one year after the introduction of canakinumab. He maintains a close follow-up with appointments every two months along with blood testing to evaluate any fluctuations of CRP, ESR, or ferritin that, along with any clinical signs, may point to a disease flare.

## Discussion

AOSD is a very rare disease [1], and myocarditis is a very uncommon and life-threatening complication, seldomly described. A Turkish case series of 84 patients with AOSD reported no cases of myocarditis [2]. Larger case series reported an AOSD myocarditis prevalence of 5%-7% [3,4]. Néel et al. stated that cardiocirculatory failure, due to myocarditis (or cardiac tamponade), is the second leading cause of admission to an intensive care unit for AOSD patients, mainly during the inaugural episode [5]. It also outlined that an accurate diagnosis and treatment initiation had a median delay of three weeks. This delay was noted in our clinical case, which probably resulted in a life-threatening complication that could have been fatal if not readily identified and treated. Patients with myocarditis are more frequently young males, have higher white blood cells count (and polymorphonuclears), and have markedly high inflammatory markers and serum ferritin when compared to AOSD patients without myocarditis [1,6], everything in accordance with the clinical setting we present. Concomitant pericarditis was also present, and it was probably the cause of the chest pain the patient experienced. Pericarditis is the most frequent cardiac finding in AOSD [1,7,8], with a prevalence of up to 40% [9], probably due to the typical serosal involvement of AOSD, and a high-grade pleural effusion was also present in our patient [10]. It is important to mention that pericarditis itself seems to be a clinical predictor for the use of biologic disease-modifying anti-rheumatic drugs (bDMARDs) [8,11]. This data points out the importance of cardiac evaluation during the initial approach to patients with suspected AOSD.

Although rarely performed, endomyocardial biopsy remains the gold standard to confirm myocarditis [12]. Cardiac MRI is very sensitive and has validated diagnostic criteria for non-ischemic myocarditis [13]; therefore, it is a very reliable diagnostic tool [14]. Herein, this was the chosen modality to diagnose myocarditis and follow-up the heart involvement.

Regarding refractory disease, although myocarditis was diagnosed in a minority of patients, a retrospective study of 96 patients with AOSD found that heart involvement occurs in as many as 30% of patients with AOSD and seems to be an independent risk factor for a disease weakly responsive to corticosteroids and DMARDs or even refractory disease [4]. Gracia-Ramos et al. also reported that the majority of patients with AOSD myocarditis are not responsive to corticosteroids alone, whereas anakinra has efficacy of up to 89% [5,6]. There are no guidelines for the treatment of AOSD since there is a lack of randomized controlled trials in adults. However, extrapolation from studies in systemic juvenile idiopathic arthritis [15,16] leads to anakinra and tocilizumab being increasingly used, as they seem to be effective in treating AOSD, even in refractory cases [8,17,18]. As such, besides being a marker of severe disease, cardiac involvement may help to identify patients where bDMARDs will eventually be needed to treat the disease [4,8]. Despite being on high-dose corticosteroids, our patient relapsed with pleuritic involvement, acute respiratory syndrome, and supraventricular tachycardia. This prompted a quick therapeutic intervention with anakinra that was shown to be lifesaving, and a complete remission of symptoms was initially achieved. If readily identified and treated, myocarditis seems to have a good short-term prognosis [4,6]. However, a secondary failure to anakinra occurred as persistent arthritis with hyperferritinemia was observed.

In one-third of patients, AOSD evolves into a chronic pattern, comprised mainly of articular symptoms [1]. This form tends to be progressive, and erosive destruction of the joints may occur if not adequately controlled. If the initial severe heart involvement is a predictor of this chronic form, this is yet to be determined as more data regarding long-term follow-up is needed. A switch to tocilizumab was proposed, but joint involvement persisted and a primary failure to therapy was established. As such, a new bDMARD switch was proposed, this time to canakinumab. This interleukin-1β inhibitor has been showing promising results in achieving complete remission in AOSD [19]. We were able to reach this goal with our patient, and complete remission is now sustained for the time being (after one year of follow-up). The timing for tapering or discontinuing bDMARDs in AOSD is yet to be determined as it was reported that it may lead to relapse [20], and still, there is not enough evidence regarding when this approach should be considered. The patient should maintain a close follow-up in order to detect any early signs of relapse, allowing a fast intervention.

## Conclusions

In conclusion, AOSD is a very rare multisystem disease that can prove to be difficult to manage. Although myocarditis is a rare complication, heart involvement can be a common initial presentation (e.g., pericarditis), making cardiac evaluation important for the initial approach. While there is no way to predict the course of the disease, a severe initial presentation with heart involvement may foretell a refractory disease. Therapy for severe cases must be quickly implemented in order to be lifesaving and avoid major sequelae. Anti-IL1 and anti-IL6 therapies seem to be the most effective for AOSD with visceral involvement and seem to be effective in maintaining disease remission.
